# A Systematic Review of Serum γ-Glutamyltransferase as a Prognostic Biomarker in Patients with Genitourinary Cancer

**DOI:** 10.3390/antiox10040549

**Published:** 2021-04-01

**Authors:** Kosuke Takemura, Philip G. Board, Fumitaka Koga

**Affiliations:** 1 Department of Urology, Tokyo Metropolitan Cancer and Infectious Diseases Center Komagome Hospital, Tokyo 113-8677, Japan; 2 ACRF Department of Cancer Biology and Therapeutics, Group of Molecular Genetics, John Curtin School of Medical Research, Australian National University, Canberra, ACT 2601, Australia; Philip.G.Board@anu.edu.au

**Keywords:** biomarkers, γ-glutamyltransferase, glutathione, prognosis, urologic neoplasms

## Abstract

γ-Glutamyltransferase (GGT), a membrane-bound enzyme, contributes to the metabolism of glutathione (GSH), which plays a critical physiological role in protecting cells against oxidative stress. GGT has been proposed as a biomarker of carcinogenesis and tumor progression given that GGT activity is important during both the promotion and invasion phases in cancer cells. Moreover, GGT expression is reportedly related to drug-resistance possibly because a wide range of drugs are conjugated with GSH, the availability of which is influenced by GGT activity. While serum GGT activity is commonly used as a quick, inexpensive, yet reliable means of assessing liver function, recent epidemiological studies have shown that it may also be an indicator of an increased risk of prostate cancer development. Moreover, elevated serum GGT is reportedly an adverse prognostic predictor in patients with urologic neoplasms, including renal cell carcinoma, prostate cancer, and urothelial carcinoma, although the background mechanisms have still not been well-characterized. The present review article summarizes the possible role of GGT in cancer cells and focuses on evidence evaluation through a systematic review of the latest literature on the prognostic role of serum GGT in patients with genitourinary cancer.

## 1. Introduction

γ-Glutamyltransferase (GGT) is one of the enzymes involved in the γ-glutamyl cycle, which contributes to the synthesis and degradation of glutathione (GSH) [1]. GSH is known to be a major intracellular water-soluble antioxidant playing a protective role against reactive oxygen species (ROS) [2]. GGT also has a possible, albeit controversial, role in the transportation of amino acids across cellular membranes [3]. In addition to being used as a hepatobiliary biomarker that is especially sensitive to excessive alcohol consumption [4], elevated serum GGT has been shown to be an adverse prognostic factor in patients with life-threatening diseases. A recent study reported on the utility of the serum GGT level as a predictive biomarker of clinical outcomes in patients with a multitude of conditions, including cardiovascular diseases, diabetes mellitus, and metabolic syndrome [4]. Moreover, serum GGT is reportedly associated with a poor prognosis in patients with liver cancer, reflecting unfavorable clinicopathological features, including vascular invasion and the tumor burden, as demonstrated in previous meta-analyses [5,6]. Subsequently, serum GGT was also found to be a significant prognostic biomarker in patients with a variety of malignant neoplasms; however, reports of serum GGT in relation to genitourinary (GU) cancer are relatively limited, and the findings among studies of renal cell carcinoma (RCC) are sometimes contradictory depending on the metastatic status of the disease [7,8,9,10,11,12]. On the other hand, serum GGT was found to be significantly and independently associated with shorter overall survival in patients with advanced GU cancer, such as metastatic prostate cancer (PC) and advanced urothelial carcinoma (UC) [13,14]. The present article aims to summarize the physiological role of GGT in cancer cells and provide a systematic review of the literature to clarify the potential prognostic role of serum GGT in patients with GU cancer from both basic and clinical scientific perspectives.

## 2. Structure, Functions, and Expression of GGT

### 2.1. GGT Family Enzymes and Structure

There are at least eight potential full-length GGT family proteins, including GGT1, GGT2, GGT3P, GGT4P, GGT5, GGT6, GGT7, and GGT8P [15]. Of these, GGT1 and GGT5 are the only two enzymes that have been shown to be catalytically active [15,16]. Immunolabelling of these closely related enzymes in human tissues has revealed different expression patterns in different organs or even within the same tissue (e.g., GGT1 is expressed on the apical surface of the renal proximal tubules whereas GGT5 is expressed in the interstitial cells of the kidney), resulting in access to different substrates [17]. Moreover, their enzymatic activities are not at the same level according to a previous kinetic analysis, which demonstrated that GGT1 is able to cleave GSH approximately 46 times faster than GGT5 [18]. Furthermore, a genetic deficiency of the GGT1 gene was found to cause severe growth failure or skeletal abnormalities while null mutants of GGT5 gene have not been associated with any obvious phenotypic changes, indicating that GGT1 functions cannot be compensated by those of GGT5 [19,20,21]. The predominance of GGT1 among the GGT family proteins in terms of GSH metabolism is further supported by a recent genetic analysis of two siblings with a GGT deficiency (i.e., glutathionuria, OMIM 231950) [22]. Whole-genome sequencing identified a large homozygous intragenic deletion in GGT1 causing glutathionuria in the patients. Other potentially active enzymes in the GGT family include GGT7, which shares amino acid similarities with GGT1. However, neither its expression pattern nor its functions have been determined [15]. Reports on the protein expression analysis of GGT7 are only limited to ovarian cancer and glioblastoma and have produced conflicting results; in the former, strong GGT7 expression was associated with higher serum GGT, which was in turn associated with an advanced tumor stage and a worse prognosis while, in the latter, strong GGT7 expression was associated with a better prognosis [23,24]. Further studies are needed to clarify the behavior of these proteins, given that GGT7 may have different enzymatic functions as it shares only 47% and 52% of its amino acid sequence with GGT1 and GGT5, respectively, and has high variation in its light chain ^15^. Taken together, GGT1 is the most extensively studied catalytic enzyme among the GGT family proteins and is ubiquitously expressed in the human body [25].

A recombinant human GGT1 protein was identified as a heterodimer consisting of two glycosylated subunits with a mean molecular mass of 80 kDa and 29 kDa, respectively. The crystal structure of human GGT1 was subsequently obtained by high-resolution X-ray crystallography [26,27]. The active site of human GGT1 was identified as Thr-381, which can be blocked by competitive inhibitors, including acivicin, azaserine, and 6-diazo-5-oxo-norleucine, all of which are glutamate analogs [28].

### 2.2. Functions of GGT

GGT is an ectoenzyme in the cellular membrane and is known to cleave the γ-glutamyl bond of most γ-glutamyl peptide substrates, including GSH, glutathione S-drug conjugates, and leukotriene C4, resulting in a broad range of protective activities against oxidative stress, drugs, and inflammation [29,30,31]. Among the molecules cleaved by GGT is GSH, a tripeptide consisting of glutamate, cysteine, and glycine that functions as a major intracellular water-soluble antioxidant [2]. Glutamate is linked via a peptide bond through its γ-carboxyl to cysteine. This unusual peptide bond makes GSH resistant to degradation by most peptidases. Among its three constituent amino acids, cysteine is the least available, and thus GGT and the γ-glutamyl cycle play an important role in the metabolism of GSH, especially under cysteine-limited circumstances [32]. In fact, experimentally overexpressed GGT in melanoma cells promoted tumor growth through an intertissue flow of GSH and increased resistance to oxidative stress by recycling cysteine from the extracellular GSH [33,34]. Likewise, transfection of melanoma cells with GGT cDNA resulted in resistance to cisplatin, a major antitumor agent, presumably because of high intracellular cysteinyl–glycine levels resulting from GGT-mediated catabolism of extracellular GSH [35].

**Figure 1:**
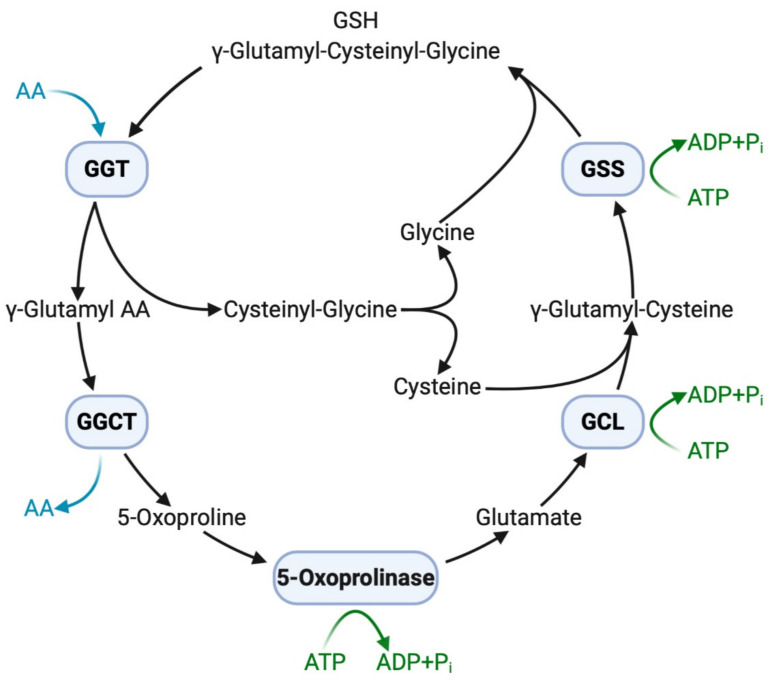
GGT and other enzymes in the γ-glutamyl cycle. Extracellular GSH (γ-glutamyl–cysteinyl–glycine) is broken down on the cell membrane by membrane-bound GGT, making component peptides available to cells with successive reactions with other enzymes. Intracellular GSH is then resynthesized, increasing resistance to oxidative stress. AA, amino acid; ADP, adenosine diphosphate; ATP, adenosine triphosphate; GCL, glutamate-cysteine ligase; GGCT, γ-glutamylcyclotransferase; GGT, γ-glutamyltransferase; GSH, glutathione; GSS, glutathione synthetase; P_i_, inorganic phosphate.

The extracellular domain of GGT cleaves the γ-glutamyl bond of GSH and releases cysteinyl–glycine and a γ-glutamyl amino acid at the initial step of the γ-glutamyl cycle (Figure 1). These peptides are subsequently cleaved by γ-glutamylcyclotransferase to yield 5-oxoproline, a free amino acid that is further converted into glutamate by the action of 5-oxoprilinase [3]. This cycle is also important for the resynthesis of intracellular GSH via step-by-step ligation of glutamate, cysteine, and glycine through the sequential action of glutamate–cysteine ligase and glutathione synthetase, leading to the homeostatic maintenance of the intracellular redox potential, as demonstrated in isolated kidney cells [2]. Another key enzyme involved in GSH homeostasis is cation transport regulator 1 (CHAC1), a newly discovered γ-glutamylcyclotransferase involved in the intracellular degradation of GSH [36]. This soluble enzyme is expressed primarily in the cytoplasm and can specifically degrade GSH into 5-oxoproline and cysteinyl-glycine but is inactive with other γ-glutamyl peptides [37]. The shared ability of GGT and CHAC1 to degrade GSH into its component peptides, including cysteine, suggests that they interactively play a scavenging function when cells are exposed to cysteine depletion [36].

**Figure 2:**
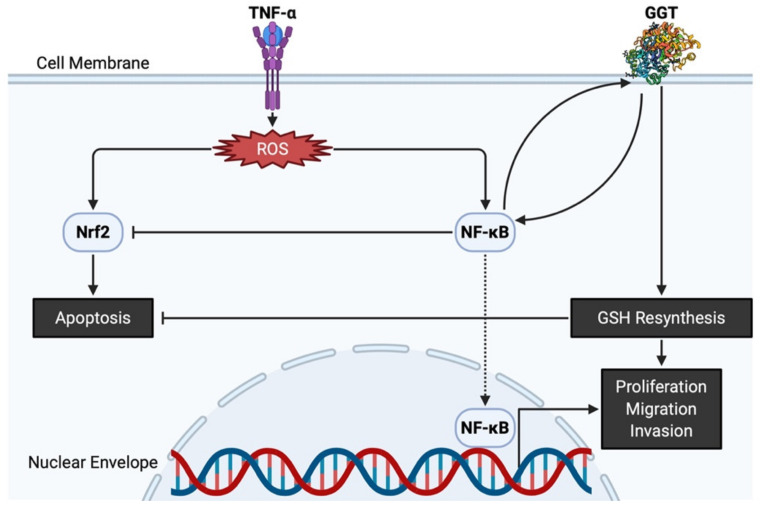
GGT and related molecules in carcinogenesis and tumor progression. TNF-α produces ROS, leading to Nrf2 and NF-κB overpresentation. GGT interacts with NF-κB and helps NF-κB interfere with apoptosis induced by Nrf2. Both nuclear translocation of NF-κB and intracellular resynthesis of GSH, each supported by the enzymatic activities of GGT, have a positive effect on the proliferation, migration, and invasion of cancer cells. GGT, γ-glutamyltransferase; GSH, glutathione; NF-κB, nuclear factor-κB; Nrf2, nuclear factor E2-related factor 2; ROS, reactive oxygen species; TNF-α, tumor necrosis factor-α.

The antioxidative properties of GGT have also been attested by evidence showing that GGT transcription is stimulated by a pro-inflammatory cytokine of tumor necrosis factor-α (TNF-α), and that various inhibitors of nuclear factor-κB (NF-κB) prevent overexpression of GGT, confirming that GGT expression induced by TNF-α is mediated by NF-κB [38]. Interestingly, GGT itself can act as a modulator of NF-κB transcriptional activity since it impairs the redox status of thiols that are indispensable for NF-κB DNA binding and gene transactivation [39]. On the other hand, nuclear factor E2-related factor 2 (Nrf2) is involved in an important signaling pathway within the antioxidant response of cancer cells to ROS [40]. Nevertheless, GGT promoter activity is reportedly independent of the Nrf2 signaling pathway, as demonstrated by transfection of cells with small interfering RNA against Nrf2 [41]. Both NF-κB and Nrf2 are induced by ROS and are believed to interfere with one another [42]. GGT located downstream of NF-κB thus promotes proliferation while avoiding apoptosis via Nrf2, eventually leading to carcinogenesis (Figure 2). Apart from its potential role in carcinogenesis, GGT expression is necessary in cell proliferation, migration, and tumor growth because GGT inhibition induces cell cycle arrest with decreased Ki67-positive cells [43]. Thus, GGT appears to be involved in both the early stages of carcinogenesis and the late stages of tumor progression.

### 2.3. Expression of GGT in Normal Cells and Cancer Cells in Urogenital Organs

The physiological function of GGT has been described most clearly in the kidney, where GGT localized on the luminal surface of the proximal tubule cells prevents GSH excretion into the urine by initiating the cleavage of GSH into its constituent amino acids, which can then be reabsorbed [25]. Positive immunostaining for GGT was also observed in secretory epithelial cells of the prostatic ducts and acini although the underlying basal epithelial cells were negative for GGT [44]. GGT expression in the urinary tract is very low and only focal expression has been observed in the stroma under the urothelium [25]. Nonetheless, increased expression of GGT was reported in experimentally induced UC in rat urinary bladder treated with the carcinogen N-butyl-N-4-hydroxybutylnitrosamine, suggesting a more direct relationship with carcinogenesis in GGT than in other enzymes involved in drug metabolism [45]. Overexpression of GGT is not a specific marker for UC but is universally observed in tumors arising from different urogenital organs, including the urethra, prostate, and kidney, according to a previous comprehensive immunohistochemical analysis using an affinity-purified polyclonal antibody against peptides corresponding to the C-terminus of the heavy subunit of human GGT [46].

In general, malignant cells maintained the same phenotype as their normal counterparts in terms of GGT expression; most of the cancer types derived from GGT-positive organs were strongly positive for GGT. Indeed, PC arising from the GGT-positive secretory epithelial cells was positive for GGT while benign prostatic hyperplasia showed weak apical expression of GGT [44]. A partial explanation for the overexpression of GGT in PC lies in the fact that androgens can elevate GGT mRNA expression under the regulation of polyomavirus enhancer activator 3, a transcriptional factor, resulting in 500–800-fold greater GGT activity in normal seminal plasma and prostatic fluid than in normal serum [47,48]. In the aforementioned comprehensive immunohistochemical analysis, clear cell RCC arising from GGT-positive proximal renal tubule epithelial cells was strongly GGT-positive with prominent membranous staining patterns [25]. A more recent immunohistochemical analysis using surgically resected RCC specimens demonstrated that 57 out of 65 cases (not including chromophobe RCC) showed moderate to strong GGT expression regardless of their Fuhrman grade [11]. On the other hand, chromophobe RCC, hypothetically arising from GGT-negative distal renal tubule epithelial cells, was completely negative for GGT in contrast to clear cell RCC [25]. In accordance with GGT protein expression, GGT mRNA expression was found to be > 100-fold lower in chromophobe RCC than in normal kidney and clear cell RCC [49]. These findings suggested that GGT expression levels in GU cancer, as in other organs, reflects the phenotype of the original cell types.

## 3. Potential Clinical Application of GGT Inhibitors

The present section focuses on the possible clinical application of targeted treatment strategies against GGT utilizing several competitive and uncompetitive inhibitors.

### 3.1. Competitive GGT Inhibitors

Acivicin, a glutamine analogue, inhibits the catalytic activities of GGT [50] and significantly decreases both the rate of intracellular GSH replenishment and the maximum intracellular GSH content [33]. Following the discovery of acivicin, several in vitro and in vivo studies demonstrated acivicin’s therapeutic potential against various types of cancer, including PC, colon cancer, and melanoma [47,51,52]. During the 1980s and 1990s, acivicin was tested for its clinical relevance in phase I and phase II trials in patients with various advanced malignancies [53,54,55,56,57,58,59,60]. Despite the objective antitumor activity observed in some trials, acivicin failed to be approved for clinical use because of the potential for severe toxicity, such as lethal myelosuppression and neurotoxicity, although the causes of these adverse events were not clearly known at the time. However, the detailed mechanism of growth inhibition by acivicin has recently been confirmed based on proteomic profiling of cancer cells. The present study demonstrated that acivicin inhibits aldehyde dehydrogenase 4A1 activity by binding to its catalytic site and does not exert its clinical effects through GGT inhibition, as acivicin has a very low affinity for human GGT [61]. Other glutamine analogues (e.g., the serine–borate complex and γ-phosphono diester analogues of glutamate) and glutamate derivatives or their analogues (e.g., sulfur derivatives of L-glutamic acid and 6-diazo-5-oxo-norleucine) have also been proposed; however, they cause similar toxicity-related problems because they not only inhibit GGT activity but also other essential glutamine metabolizing enzymes, including glutaminases and L-asparagine synthetase [62,63,64,65].

### 3.2. Uncompetitive GGT Inhibitors

Several novel inhibitors have been developed to overcome the low affinity and high toxicity of existing GGT inhibitors. OU749, a lead compound, is an uncompetitive inhibitor that occupies the acceptor site but not the γ-glutamyl site of GGT [66]. It is worth noting that OU749 is more than 150-fold less toxic than acivicin but has 7–10-fold greater inhibitory potency against GGT isolated from human kidney than GGT isolated from rat or mouse kidney. This ability of OU749 has also been validated under in vivo conditions using GGT-positive cell lines; OU749 blocked GGT catabolism of GSH, providing a basis for further development of a novel therapeutic agent capable of inhibiting GGT via a mechanism distinct from that of the toxic glutamine analogue [67]. Ovothiols, which are histidine-derived thiols isolated from sea urchin eggs, are known to protect the eggs from high oxidative stress at fertilization and may induce autophagy-dependent cell death in human hepatic cancer cells with high GGT expression while leaving normal cells unaffected [68]. Current studies have revealed that the uncompetitive GGT-inhibitory properties of ovothiols produced by marine invertebrates provide great promise for improving molecular-targeted therapy for GGT-positive malignancies, including GU cancer [69,70]. Clinical trials and optimization of these less toxic GGT inhibitors are warranted for the development of novel treatments.

## 4. Elevation of Serum GGT in Patients with GU Cancer

### 4.1. Sources of Serum GGT

Even though serum GGT measurements are routinely used as a clinical laboratory test to detect liver dysfunction, bile duct conditions, and alcohol consumption-related damage^4^, it is unknown whether the elevated serum GGT derives from the same source in hepatobiliary diseases, nor is there direct evidence that serum GGT derives from the liver since increased hepatic GGT is apparently unnecessary to elevate the serum GGT level [71]. However, previous studies shed light on possible GGT sources in tumor tissues based on the fact that various cultured human cancer cell lines of different origins, including PC, were found to release soluble GGT corresponding to a specific GGT fraction, namely big-GGT (eluting between 10.0 and 13.5 mL in gel filtration, the molecular weight > 2000 kDa), which occurs in the plasma of healthy individuals [72,73]. Additionally, the enzymatic activity of GGT in the supernatant of culture media was found to increase in parallel with the cell growth of all the cell lines tested in the study [73]. These findings support the notion that not only hepatic cells but also cancer cells originating from a variety of organs can be an important source of serum GGT. Comparative studies of human systemic serum GGT levels and local GGT expression levels are, however, very scarce irrespective of cancer type. Only three studies have investigated this relationship using clinical laboratory data and immunohistochemical analysis of pathological specimens [11,23,74]. Notably, serum GGT significantly correlated with tissue GGT in patients with RCC, which significantly decreased after nephrectomy in patients with elevated preoperative serum GGT 11. Likewise, elevated serum GGT was associated with higher tumor GGT expression and an advanced tumor stage in ovarian cancer [23]. These findings suggest that serum GGT may reflect the tumor burden in patients with tumors overexpressing GGT. In contrast, in patients with gastric cancer the opposite results were found, indicating peripheral clearance of GGT in specific types of cancer [74]. Further investigation is needed to clarify the determinants of the serum GGT level.

### 4.2. Elevation of Serum GGT in Patients with GU Cancer

Serum GGT is associated with cancer mortality in both men and women yet, to date, few studies with an adequate sample size and follow-up data have investigated the association between serum GGT and cancer incidence [75]. Population-based studies have been conducted for PC but not for other GU cancer. Among these studies, a large prospective study in Sweden enrolling 545,460 patients demonstrated that elevated serum GGT was linked to an increased overall cancer risk in both men and women, and that patients with a diagnosis of PC with elevated serum GGT had an increased risk of a secondary diagnosis of a primary tumor, suggesting the possibility of shared aspects of GGT-related metabolism in PC and other later malignancies [76,77]. Similarly, elevated serum GGT was positively and independently associated with the risk of PC in a Finnish cohort of 2390 men aged 42–61 years over long-term follow-up [78]. Although no comparative study of clinical outcomes of PC according to serum GGT levels has been performed, previous studies suggested that overexpression of GGT in PC cells may be associated with an aggressive phenotype [79]. In RCC, 70% and 12% of patients with metastatic RCC and localized RCC showed serum GGT > 60 U/L, respectively, indicating the sensitivity of serum GGT as a marker for predicting the presence of a metastasis [80]. Another study also revealed that serum GGT increased to a greater degree in RCC patients with non-bone metastases than in those with non-metastatic RCC 7. To the best of our knowledge, no population-based study of serum GGT and its relationship with the risk of UC development has ever been conducted.

### 4.3. Serum GGT as a Prognostic Biomarker in Patients with Cancer

Hitherto, elevated serum GGT has been studied extensively as an unfavorable prognostic biomarker in patients with liver cancer, including hepatocellular carcinoma and intrahepatic cholangiocarcinoma, on which dozens of clinical studies have been conducted. Recently, two meta-analyses demonstrated that patients with a high serum GGT level not only had a poor prognosis (in terms of overall survival, recurrence-free survival, and disease-free survival) but also had several unfavorable clinicopathological features, including vascular invasion and the tumor burden [5,6]. Similar findings were also reported in other organs, including the uterus [81,82,83,84,85], colorectum [86], ovaries [23], esophagus [87,88], breasts [89,90], gallbladder [91], nasopharynx [92,93], stomach [74,94], lungs [95], and pancreas [96]. In urogenital organs, too, recent studies have demonstrated that serum GGT can predict survival in patients with advanced GU cancer, including RCC [11,12], PC [13], and UC [14]. Thus, it can be speculated that serum GGT has the potential to become a general biomarker of the tumor burden in patients with GU cancer, given that tumors arising from the urogenital organs overexpress GGT [46], possibly leading to its release into the bloodstream and its presence in the serum of patients with a metastatic disease in particular. However, little is known about patients with GU cancer because no systematic reviews on this topic have been done. The following section presents a systematic review and synthesis of the currently available evidence.

## 5. A Systematic Review of Serum GGT in Patients with GU Cancer

### 5.1. Study Selection

**Figure 3:**
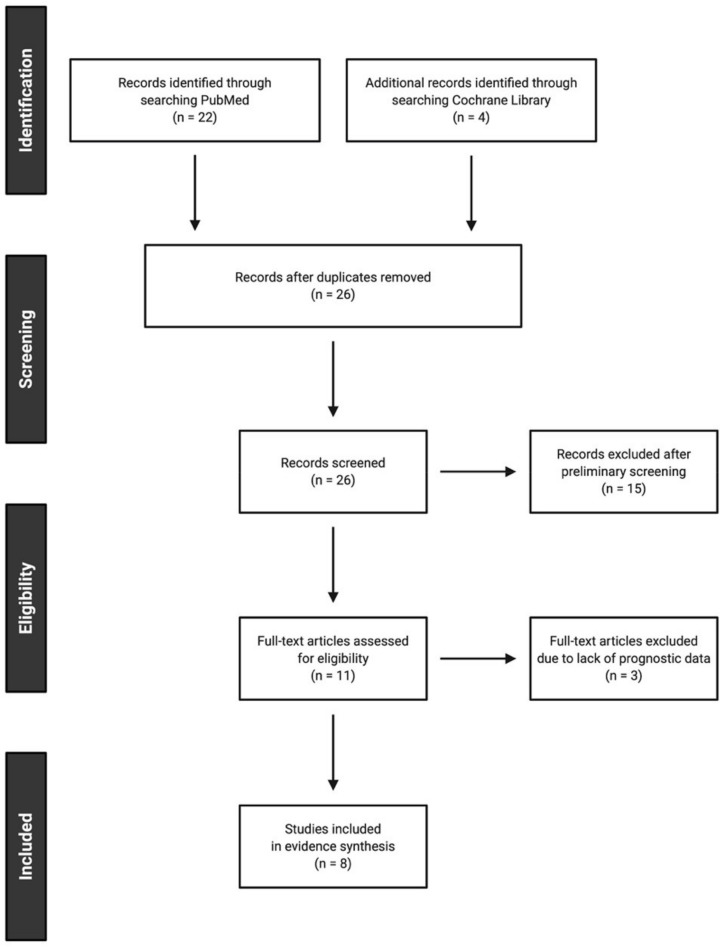
Flow diagram of the literature search and article selection in accordance with the preferred reporting items for systematic reviews and meta-analyses statement.

Thus far, only a few studies have investigated the prognostic role of serum GGT in patients with urologic neoplasms, including RCC, PC, and UC. A systematic search of articles written in English was done on 31 December 2020 using PubMed and the Cochrane Library using the search terms, (gamma-glutamyltrans* AND (prognos* OR survival) AND (“renal cell carcinoma” OR “prostate cancer” OR “urothelial carcinoma” OR “transitional cell carcinoma”)). Two independent investigators (K.T. and F.K.) conducted the search and selection of articles. Potential discrepancies were resolved by open discussion. In total, 26 articles were extracted, from which eight relevant articles published between August 2014 and November 2020 were identified in accordance with the preferred reporting items for systematic reviews and meta-analyses statement [97]. Details of the search and article selection are summarized in the flow diagram (Figure 3). A PICO framework (i.e., participants, intervention, comparator, and outcomes) was applied to the screening as well as the interpretation process in the systematic review [98]. In brief, clinical studies were considered eligible if they fulfilled the following criteria:

(P): Participants in the study had non-metastatic or metastatic GU cancer.

(I): Surgical and/or systemic interventions were given.

(C): Patients were compared in terms of their pre-therapeutic serum GGT levels.

(O): Prognostic outcomes, such as survival rate and/or hazard ratio, were reported.

Only original articles investigating the prognostic role of serum GGT in GU cancer were included. Review articles, case reports, editorial comments, letters, meeting abstracts, and studies not published in English were excluded.

### 5.2. Study Characteristics

In total, 26 articles were identified, from which 15 and 3 were excluded by title/abstract and full-text screening, respectively, as per the PICO framework. Finally, eight studies were included in the analysis; six were studies of RCC, one was a study of PC, and one was a study of UC (Table 1). Most of the studies on RCC were conducted with patients receiving surgery while the remaining studies were conducted with patients with advanced GU cancer treated systemically (e.g., using tyrosine kinase inhibitors or nivolumab for metastatic RCC, enzalutamide for metastatic PC, and chemotherapy for advanced UC). It is worth noting that the results of the six studies of RCC were often contradictory; one study enrolling a relatively small patient cohort with heterogeneous clinicopathological demographics (*n* = 80) showed that elevated serum GGT was observed only in patients with RCC with non-bone metastases but did not predict the survival of the patients [7] while another study enrolling 700 patients with non-metastatic RCC revealed that serum GGT predicted metastasis-free survival but not overall survival or cancer-specific survival [9]. In contrast, the remaining four studies of RCC that included patients with metastatic RCC or RCC with a venous tumor thrombus demonstrated a significant association between the pre-therapeutic serum GGT level on both univariable and multivariable analyses [8,10,11,12]. Studies of patients with metastatic PC or advanced UC also revealed that elevated serum GGT was independently associated with shorter overall survival [13,14].

### 5.3. Evidence Synthesis

**Figure 4:**
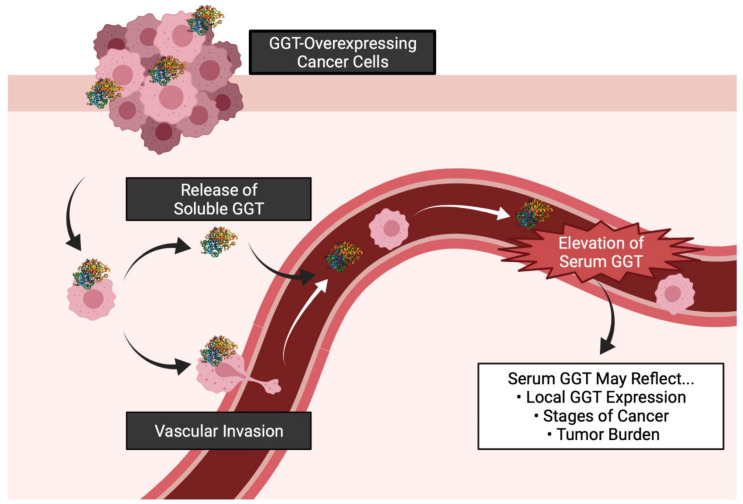
Possible mechanisms underlying serum GGT elevation in patients with GGT-overexpressing cancer cells. Cancer cells may either release GGT or directly invade blood vessels while overexpressing GGT on their cell membrane. GGT can be detected in the peripheral blood of patients especially when cancer cells have high GGT expression, the disease is advanced, or the tumor burden is heavy. GGT, γ-glutamyltransferase.

Elevated serum GGT was more predictive of the prognosis of advanced diseases [8,10,11,12,13,14] than of early stage diseases [7]. It may therefore be hypothesized that the heavy tumor burden is required for GGT overexpression by cancer cells to become detectable in the serum of patients (Figure 4).

In fact, the pre-therapeutic serum GGT level was significantly associated with the prostate-specific antigen level at the initiation of enzalutamide therapy, suggesting that GGT may increase together with an increase in the tumor burden [13]. Moreover, a subset of RCC patients with elevated GGT in preoperative serum samples showed a significant decrease in the post-operative serum GGT level [11]. Furthermore, early changes in serum GGT levels during the first two months of nivolumab therapy as well as the baseline serum GGT level were significantly associated with survival in patients with metastatic RCC [12]. An important question is whether the level of systemic serum GGT directly reflects the level of local GGT expression in cancer patients. A previous immunohistochemical analysis of ovarian cancer corroborated this association [23]. Similarly, a study of gastric cancer further suggested a joint effect between serum and tissue GGT levels [74]. In addition, an immunohistochemical analysis of RCC revealed that preoperative serum GGT levels significantly correlated with tissue GGT levels which were semi-quantified by the intensity of immunohistochemical staining (i.e., negative to weak, moderate, and strong) at the respective median serum GGT levels of 29, 48, and 109 U/L (*p* = 0.004) [11]. Comparison of serum and tissue GGT levels in patients with GU cancer is, however, very limited, and to the best of our knowledge, no such studies have been done in patients with PC or UC.

Serum exosomal GGT activity has recently been reported as superior to serum total GGT activity as a biomarker for predicting the clinicopathological features of patients with RCC [99]. The authors of the study concluded that exosomal GGT reflected the characteristics of the original cancer cells and might indeed predict the likelihood of a microvascular invasion while the total serum GGT activity could not. Further comparative studies are required to characterize the interactive impact of serum and tissue GGT levels on the prognosis of patients with GU cancer. The obtainment of each of these results from a single institution in a retrospective manner may have introduced a bias stemming from the unique patient demographics or preferred treatment strategies at the respective institutions. Nevertheless, serum GGT might well become universally accepted as a prognosticator in certain patients with GU cancer. Another possible limitation of the present study is the unavailability of kinetic data on serum GGT apart from two studies of RCC, which assessed relative changes in serum GGT at a single time point after either radical nephrectomy or nivolumab therapy [11,12]. The final limitation is the sparsity of data on PC and UC; however, existing evidence supports the notion that serum GGT is significantly and independently associated with overall survival in patients with advanced malignancies [13,14]. Further studies are needed to enhance the clinical utility of serum GGT in the context of disease status in patients with GU cancer.

## 6. Conclusions

We discussed the physiological functions of GGT at the molecular level and its overexpression in a variety of malignancies. GGT is indispensable for cancer cells, which utilize extracellular GSH as a source of intracellular cysteine to synthesize GSH to acquire resistance to oxidative stress. Moreover, a systematic review of clinical studies on the prognostic significance of serum GGT in patients with GU cancer suggested a possible association of serum GGT with tissue expression, stages of cancer, and the tumor burden. Further research is required to identify the precise role of serum GGT and explore the utility of its kinetics as a prognostic biomarker in patients with GU cancer. Furthermore, less toxic uncompetitive inhibitors of GGT, such as OU749 and ovothiols, have the potential to become therapeutic agents for targeted therapy in patients with tumors with GGT-overexpression [67,68,69,70]. Future prospective clinical trials of GGT blockade therapies are warranted.

## Figures and Tables

**Table 1:** Previous literature on serum γ-glutamyltransferase as a prognosticator in patients with genitourinary cancer.

Author (Year)	Country	Participants (P)	Intervention (I)	Comparator (C), Cut-off of Serum GGT (U/L)	Outcomes (O)	Reference
Ramankulov, A. (2007)	Germany	80 patients with non-metastatic or metastatic RCC: pN0M0, *n* = 32 (40%); pN1M0, *n* = 11 (14%); M1, and *n* = 37 (46%).	Surgery	40.6	Serum GGT increased in patients with non-bone metastases (median 25.3 U/L) in comparison to healthy controls (median 14.3 U/L). Elevated serum GGT was not a predictive factor of survival (*p* = 0.213)	[7]
Hofbauer, S.L. (2014)	Austria	921 patients with non-metastatic or metastatic RCC: pN+, *n* = 29 (3%); M1, *n* = 118 (13%).	Surgery	17.5, 34.5, and 181.5	Serum GGT was an independent prognostic factor for CSS. Serum GGT increased with advanced pathological stages, Fuhrman grade, and tumor necrosis. Adding GGT to a base model increased discrimination from 0.9% to 1.8%.	[8]
Dalpiaz, O. (2015)	Austria	700 patients with non-metastatic RCC	Surgery	40	Elevated serum GGT was associated with shorter MFS (*p* = 0.025) but not with OS (*p* = 0.108) or CSS (*p* = 0.242). Serum GGT failed to reach independent predictor status for MFS prediction.	[9]
Luo, C. (2017)	China	179 patients with non-metastatic RCC with venous tumor thrombus	Surgery	37.5	Elevated serum GGT was significantly associated with an advanced tumor stage, was a significant predictor of shorter CSS (HR 2.99, *p* < 0.001) and RFS (HR 2.59, *p* < 0.001) on univariable analysis, and was an independent prognostic biomarker on multivariable analysis.	[10]
Takemura, K. (2019)	Japan	125 patients with advanced UC. Inoperable cT4, *n* = 42 (34%); cN+, *n* = 92 (74%); cM+, *n* = 42 (34%)	Systemic chemotherapy, *n* = 93 (74%); surgery, *n* = 33 (26%); radiation, *n* = 6 (5%)	60	Elevated serum GGT at the diagnosis of advanced UC was associated with shorter OS on both univariable (HR 3.25, *p* < 0.001) and multivariable (HR 2.97, *p* < 0.001) analysis irrespective of liver metastases and hepatic comorbidities.	[14]
Takemura, K. (2019)	Japan	60 patients with metastatic PC. Regional LN, *n* = 23 (46%); non-regional LN, *n* = 3 (6%); bone, *n* = 33 (66%); other sites, *n* = 12 (24%)	Enzalutamide	40	Elevated serum GGT was associated with shorter OS (HR 2.68, *p* = 0.018) and PSA–PFS (HR 3.23, *p* = 0.002). PSA changes after enzalutamide therapy were less evident in the elevated serum GGT group than in the normal serum GGT group (−45% versus −90%, *p* = 0.049).	[13]
Takemura, K. (2020)	Japan	146 patients with metastatic RCC. cN+, *n* = 44 (30%); cM+, *n* = 143 (98%)	TKI	1.5 × ULN	Elevated serum GGT was associated with shorter OS (HR 3.89, *p* < 0.001). Preoperative serum GGT was 29, 48, and 109 U/L in patients with RCC specimens showing negative to weak, moderate, and strong GGT expression, respectively (*p* = 0.004).	[11]
Ishiyama, Y. (2020)	Japan	69 patients with metastatic RCC. A single metastasis, *n* = 24 (35%); multiple metastases, *n* = 45 (65%)	Nivolumab	49	Elevated baseline serum GGT was associated with shorter OS (*p* = 0.005) but not with PFS (*p* = 0.092). Serum GGT increase after nivolumab therapy was associated with shorter OS (*p* = 0.023) and PFS (*p* = 0.008).	[12]

CSS, cancer-specific survival; GGT, γ-glutamyltransferase; HR, hazard ratio; LN, lymph node; MFS, metastasis-free survival; OS, overall survival; PC, prostate cancer; PFS, progression-free survival; PSA, prostate-specific antigen; RCC, renal cell carcinoma; RFS, recurrence-free survival; TKI, tyrosine kinase inhibitor; UC, urothelial carcinoma; ULN, upper limit of normal.
